# Brain volumes in alcohol use disorder: Do females and males differ? A whole‐brain magnetic resonance imaging mega‐analysis

**DOI:** 10.1002/hbm.26404

**Published:** 2023-07-12

**Authors:** Eleonora Maggioni, Maria G. Rossetti, Nicholas B. Allen, Albert Batalla, Marcella Bellani, Yann Chye, Janna Cousijn, Anna E. Goudriaan, Robert Hester, Kent Hutchison, Chiang‐Shan R. Li, Rocio Martin‐Santos, Reza Momenan, Rajita Sinha, Lianne Schmaal, Nadia Solowij, Chao Suo, Ruth J. van Holst, Dick J. Veltman, Murat Yücel, Paul M. Thompson, Patricia Conrod, Scott Mackey, Hugh Garavan, Paolo Brambilla, Valentina Lorenzetti

**Affiliations:** ^1^ Department of Electronics, Information and Bioengineering Politecnico di Milano Milan Italy; ^2^ Department of Neurosciences and Mental Health Fondazione IRCCS Ca'Granda Ospedale Maggiore Policlinico Milan Italy; ^3^ Department of Neurosciences, Biomedicine and Movement Sciences, Section of Psychiatry University of Verona Verona Italy; ^4^ Department of Psychology University of Oregon Eugene Oregon USA; ^5^ Department of Psychiatry University Medical Center Utrecht Brain Center, Utrecht University Utrecht the Netherlands; ^6^ BrainPark, Turner Institute for Brain and Mental Health School of Psychological Sciences Melbourne Australia; ^7^ Monash Biomedical Imaging Monash University Melbourne Australia; ^8^ Neuroscience of Addiction Lab, Department of Psychology, Education and Child Studies Erasmus University Rotterdam the Netherlands; ^9^ Department of Psychiatry, Amsterdam Institute for Addiction Research Amsterdam UMC, University of Amsterdam Amsterdam the Netherlands; ^10^ School of Psychological Sciences University of Melbourne Melbourne Australia; ^11^ Department of Psychology and Neuroscience University of Colorado Boulder Boulder Colorado USA; ^12^ Department of Psychiatry and of Neuroscience Yale University School of Medicine New Haven Connecticut USA; ^13^ Department of Psychiatry and Psychology, Hospital Clinic, IDIBAPS, CIBERSAM and Institute of Neuroscience University of Barcelona Barcelona Spain; ^14^ Clinical NeuroImaging Research Core, Office of the Clinical Director National Institute on Alcohol Abuse and Alcoholism Bethesda Maryland USA; ^15^ Department of Psychiatry Yale University School of Medicine New Haven Connecticut USA; ^16^ Orygen Parkville Australia; ^17^ Centre for Youth Mental Health The University of Melbourne Melbourne Australia; ^18^ School of Psychology and Illawarra Health and Medical Research Institute University of Wollongong Wollongong Australia; ^19^ Australian Characterisation Commons at Scale (ACCS) Project Monash eResearch Centre Melbourne Australia; ^20^ Department of Psychiatry VU University Medical Center Amsterdam the Netherlands; ^21^ Imaging Genetics Center, Mark and Mary Stevens Neuroimaging and Informatics Institute Keck School of Medicine, University of Southern California Los Angeles California USA; ^22^ Department of Psychiatry Universite de Montreal, CHU Ste Justine Hospital Montreal Canada; ^23^ Department of Psychiatry University of Vermont Burlington Vermont USA; ^24^ Department of Pathophysiology and Transplantation University of Milan Milan Italy; ^25^ Neuroscience of Addiction and Mental Health Program, Healthy Brain and Mind Research Centre, School of Behavioral and Health Sciences Faculty of Health Sciences, Australian Catholic University Fitzroy Victoria Australia

**Keywords:** alcohol dependence, alcohol use disorders, MRI, sex, voxel‐based morphometry

## Abstract

Emerging evidence suggests distinct neurobiological correlates of alcohol use disorder (AUD) between sexes, which however remain largely unexplored. This work from ENIGMA Addiction Working Group aimed to characterize the sex differences in gray matter (GM) and white matter (WM) correlates of AUD using a whole‐brain, voxel‐based, multi‐tissue mega‐analytic approach, thereby extending our recent surface‐based region of interest findings on a nearly matching sample using a complementary methodological approach. T1‐weighted magnetic resonance imaging (MRI) data from 653 people with AUD and 326 controls was analyzed using voxel‐based morphometry. The effects of group, sex, group‐by‐sex, and substance use severity in AUD on brain volumes were assessed using General Linear Models. Individuals with AUD relative to controls had lower GM volume in striatal, thalamic, cerebellar, and widespread cortical clusters. Group‐by‐sex effects were found in cerebellar GM and WM volumes, which were more affected by AUD in females than males. Smaller group‐by‐sex effects were also found in frontotemporal WM tracts, which were more affected in AUD females, and in temporo‐occipital and midcingulate GM volumes, which were more affected in AUD males. AUD females but not males showed a negative association between monthly drinks and precentral GM volume. Our results suggest that AUD is associated with both shared and distinct widespread effects on GM and WM volumes in females and males. This evidence advances our previous region of interest knowledge, supporting the usefulness of adopting an exploratory perspective and the need to include sex as a relevant moderator variable in AUD.

## Graphical abstract

We aimed to characterize the sex differences in gray matter and white matter correlates of alcohol use disorder using a whole‐brain, voxel‐based, multi‐tissue mega‐analytic approach. We found that individuals with AUD relative to controls had lower GM volume in widespread brain clusters. Group‐by‐sex effects were found in cerebellar GM and WM volumes, which were more affected by AUD in females than males.

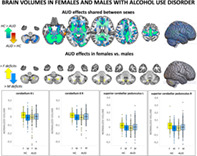

## 1 INTRODUCTION

Alcohol use disorders (AUD), including alcohol dependence, now affect around 283 million people worldwide. Most of those with AUD are males, yet females represent a substantial proportion (~15%) (Organization, 2019). This proportion is also expected to grow, due to the significant increase in the rate of yearly alcohol consumption observed in females but not in males (Grucza et al., 2018).

Importantly, females and males exhibit different patterns, motivations, and effects of drinking (Priddy et al., 2017). Males have a faster progression from first drink to dependence (Keyes et al., 2010), more frequent reward‐related drinking (Glockner‐Rist et al., 2013), and higher risk of alcohol‐related behavioral problems (Erol & Karpyak, 2015) than females. Conversely, females are more susceptible to stress‐related drinking (Peltier et al., 2019), alcohol‐related psychological distress (Jeong et al., 2019), and comorbid psychiatric or physical conditions, including depression (Erol & Karpyak, 2015; Procopio et al., 2013), than males.

While sex differences are apparent in many aspects of AUD, the underlying mechanisms are unclear. Emerging evidence from magnetic resonance imaging (MRI) studies shows sex differences in brain volume abnormalities in samples with AUD, but the findings are mixed.

Besides the acknowledged general influences of AUD on prefronto‐striatal‐limbic regions (Mackey et al., 2019; Xiao et al., 2015; Yang et al., 2016) and their structural connections (Spindler et al., 2022), larger effects of AUD in females compared to males were reported for total gray matter (GM) and white matter (WM) volumes (Agartz et al., 2003; Hommer et al., 2001; Mann et al., 2005; Momenan et al., 2012; Monnig et al., 2013). Of note, alcohol‐by‐sex effects on global brain measures were reported by Pfefferbaum et al. (2001) but not confirmed by other studies (Demirakca et al., 2011; Sawyer et al., 2017).

Larger effects of AUD in males compared to females were reported for regional brain measures, including thickness and volume of the insular cortex (Demirakca et al., 2011; Momenan et al., 2012) and volume of the corpus callosum, but without alcohol‐by‐sex effects (Ruiz et al., 2013). Conversely, alcohol‐by‐sex effects were reported for the left orbitofrontal cortical thickness, which was more affected by alcohol in females compared to males (Thayer et al., 2016), and for the amygdala (Grace et al., 2021; Rossetti et al., 2021) and reward network volumes (Fein et al., 2013; Sawyer et al., 2017), which were more affected by alcohol in males compared to females.

Overall, inconsistent findings on sex differences in the neuroanatomy in individuals with AUD may be due to the limited number of studies on this topic and their methodological limitations. First, most studies are characterized by small samples (<100 subjects), which limited the statistical power to detect more subtle alcohol‐related effects on brain anatomy (Agartz et al., 2003; Mechtcheriakov et al., 2007). Second, these samples are typically heterogeneous in their clinical characteristics, comprising individuals with abuse, heavy use, and dependence, either abstinent or not (e.g., Fein et al., 2013; Thayer et al., 2016) limiting the specificity of the obtained results. In this respect, it should be noticed that alcohol abuse and dependence were recently grouped in the AUD DSM‐5 classification but differ in terms of drinking consequences (Agrawal et al., 2011); likely, abstinence is a broad concept that refers to a state of non‐engagement in drinking behaviors (Fernandez et al., 2020).

Third, the neuroanatomical findings are generated using volume‐based (e.g., Demirakca et al., 2011; Mechtcheriakov et al., 2007; Sawyer et al., 2017) and/or surface‐based (Momenan et al., 2012; Thayer et al., 2016) approaches, as well as using statistical models that vary across studies (either including or excluding age, intracranial volume, etc.). Fourth, most studies focus on selected brain regions of interest (ROIs) (e.g., Agartz et al., 2003; Demirakca et al., 2011; Sawyer et al., 2017) which are largely informed by theoretical models of substance dependence based mostly on male samples. In addition, ROI approaches often rely on pre‐defined parcellation schemes, which are not optimized for each application, and cannot detect subtle differences within (or across) the ROIs that might represent subtle markers of AUD.

The present study aimed to examine sex differences in the local brain anatomy of alcohol dependence (which will be referred to as AUD), and severity of use in affected subjects, while addressing limitations of the existing literature. We performed a mega‐analysis on a large sample (979 individuals) from the ENIGMA Addiction Working Group (WG), including 653 individuals with a diagnosis of AUD (222 females and 431 males) and 326 controls (99 females and 227 males), to provide adequate statistical power to detect potential effects of AUD.

This study fits into the ENIGMA Addiction research works that explored alcohol‐by‐sex effects on brain anatomy; using the large sample made available from the consortium, our study aimed to extend previous surface‐based ROI findings by adopting a volume‐based exploratory perspective. As compared with previous ENIGMA Addiction studies within this research line, this study adopted a largely overlapping sample but a different methodology to respond to a similar but broader clinical question, that is, to identify shared and distinct effects of AUD on whole‐brain GM and WM volumes between females and males. A whole‐brain, voxel‐based, interaction approach was used to map alcohol‐by‐sex effects on GM and WM volumes in clusters that do not necessarily correspond with pre‐defined ROI boundaries. Specifically, we performed voxel‐based morphometry (VBM) comparisons on GM and WM volume maps, in which the intensity of each voxel represents the absolute amount of tissue (i.e., volume) in the voxel. While the VBM approach enabled an unbiased voxel‐by‐voxel exploration of brain tissue volumes in AUD, the joint investigation of GM and WM allowed, for the first time, the detection of subtle alcohol‐by‐sex alterations occurring at the GM‐WM interface. Furthermore, the WM volume analysis provided information on WM macrostructural characteristics that could complement the more extensive knowledge of WM microstructural integrity in AUD provided by DTI studies.

Based on the emerging evidence to date, we hypothesized that (i) AUD would be associated with GM and WM volume reductions in regions of the memory and reward circuits (e.g., basal ganglia, thalamus, hippocampus, dorsolateral prefrontal cortex, and anterior cingulate cortex) (Mackey et al., 2019; Xiao et al., 2015; Yang et al., 2016); (ii) females would show greater volume reductions induced by AUD than males in prefrontal and default mode network GM regions and WM connections, which might underlie the higher co‐occurrence of depressive symptoms in AUD females than males (Hamilton et al., 2015; Procopio et al., 2013), and (iii) males would show greater volume reductions induced by AUD than females in reward network GM regions and WM connections, which might explain the more pronounced reward craving tendency in AUD males than females (Glockner‐Rist et al., 2013).

## 2 MATERIALS AND METHODS

### 2.1 Participants

Socio‐demographic, substance use, and T1‐weighted brain MRI data were collected from 1348 participants across 10 Clinical Research Centers participating in the ENIGMA Addiction Working Group. All participants provided written informed consent to the protocol, which was approved by the relevant local ethics committees. The original dataset was screened by excluding individuals with (i) lifetime or current primary psychiatric disorders other than AUD, (ii) current dependence on substances other than alcohol, (iii) current abstinence from alcohol for more than 30 days. We also excluded individuals with intelligence quotient (IQ) < 80, missing demographic or education information or low MRI data quality. The dataset selected for statistical analyses included 979 participants—653 individuals with AUD (33.91 ± 10.47 years, 222 females and 431 males) and 326 controls (30.23 ± 10.43 years, 99 females and 227 males). For comparison, previous ENIGMA Addiction research work by Rossetti et al. (2021) used a largely overlapping sample of 986 participants, with the difference that in this study we excluded 7 participants with AUD due to low quality of SPM tissue segmentation.

Diagnosis was based on DSM‐IV criteria or, in only one site, drinking frequency thresholds (i.e., heavy drinking with ≥5 drinks per occasion [males], ≥4 drinks per occasion [females] ≥ 5 times in the past month); severity of alcohol use was assessed using number of standard drinks per month (www.niaaa.nih.gov/alcohols-effects-health/overview-alcohol-consumption/what-standard-drink). Depending on the center, AUD individuals abstained from alcohol for at least 1 day and at maximum 30 days before the MRI examination. Further information on inclusion criteria, sample, and MRI characteristics of the single centers can be found in Appendix S1.

### 2.2 MRI data processing

All T1‐weighted images were processed on the same workstation using Statistical Parametric Mapping software (SPM12, https://www.fil.ion.ucl.ac.uk/spm) (Friston, 2003) and its CAT12 (http://141.35.69.218/cat/) and masking (Ridgway et al., 2009) toolboxes, in‐house scripts, and functions from the Statistics and Machine Learning Toolbox™ on Matlab R2019a (The Mathworks, Inc., Natick).

#### 2.2.1 Pre‐processing

The subjects’ T1‐weighted images were oriented to match the SPM tissue priors—by setting the origin at the anterior commissure and the bicommissural line on the horizontal axis—and segmented into brain tissues using SPM12 segmentation. Using DARTEL (Diffeomorphic Anatomical Registration Through Exponentiated Lie algebra), the subjects’ GM and WM images were warped to the corresponding group template; the resulting images were normalized to the Montreal Neurological Institute (MNI) space, smoothed with a 3D 6‐mm Full Width at Half Maximum Gaussian kernel, and modulated to preserve the total amount of signal from each region. After a visual inspection of the tissue segmentation quality, we performed the CAT12‐based quality check by (i) verifying the spatial registration across images, (ii) identifying and carefully inspecting the images with an overall intensity covariance below two standard deviations. Each subject's total intracranial volume (TIV) was estimated by summing the GM, WM, and cerebrospinal fluid images in the subject's native space. Group‐level optimal threshold GM and WM masks were computed from the pre‐processed images using the SPM12 Masking toolbox and used to delimit the brain regions for VBM comparison.

The pre‐processed images with an adequate quality (*n* = 979) were subject to a step of data harmonization across centers before the VBM statistical analyses. The data harmonization across centers was performed using the Matlab‐based ComBat tool, which aims to remove any confounding contributions of the MRI data acquisition center while conserving the variance in the data due to other (e.g., biological) factors of interest (Fortin et al., 2018). In our study, ComBat was applied to each voxel of the GM and WM images and to TIV values by using center as confounder, and age, sex, group, and years of education as factors of interest. The harmonized GM and WM images, and TIV values were then entered in the following VBM statistical analyses.

#### 2.2.2 Statistical analyses

The impact of group (AUD, control), sex (females, males), group‐by‐sex, and monthly standard drinks or cigarettes (shared and distinct between AUD females and males) on local GM and WM volumes was assessed via whole‐brain, voxel‐based comparisons of the pre‐processed GM and WM images using mass‐univariate factorial General Linear Model (GLM) designs in SPM12.

Voxel‐based GLM analyses were separately conducted on GM and WM images to examine the effects of group, sex, and group‐by‐sex on GM or WM volumes in the entire dataset (GLM #1, *n* = 979), and the impact of monthly standard drinks and monthly cigarettes—both in interaction with sex—on GM or WM volumes in AUD subjects having this information (GLM #2, *n* = 453). In all GLM designs, age (interacting with group or monthly drinks or cigarettes, as appropriate), years of education, and harmonized TIV were modelled as confounding factors. The effects of (i) group, sex, and group‐by‐sex (GLM #1), (ii) monthly standard drinks or monthly cigarettes in AUD females and/or males, and females versus males (GLM #2) on voxel‐based GM or WM volumes were assessed through nonparametric permutation inferences on the corresponding GLM *t‐*contrasts (*n* = 5000). Significance was set to *p* < .05 after peak‐based family wise error correction (FWE). A secondary threshold‐free cluster enhancement (TFCE) analysis was performed to select the most relevant clusters from the main analysis. A post‐hoc region of interest (ROI) GLM analysis was performed on the GM or WM volumes in the VBM clusters resulting from the GLM #1 group‐by‐sex contrasts. The objective of the additional ROI analysis was merely to investigate any pairwise differences among group‐by‐sex subsets that could not be assessed with the VBM design. Further details on voxel‐ and ROI‐based GLM design specification, estimation, and interrogation may be found in Appendix S1.

## 3 RESULTS

### 3.1 Socio‐demographic and substance use data

The sample demographic and substance use characteristics are reported in Table 1. Sex distribution was non‐significantly different between groups. The AUD group was older, had fewer education years and higher rates of alcohol and nicotine use than the control group. The number of monthly alcohol standard drinks was higher in males than females with AUD. Significant effects of the testing sites emerged for all behavioral variables.

**TABLE 1:** Demographic and clinical information of the sample.

	AUD		HC		Group (AUD vs. HC)		Sex (females vs. males)		Group‐by‐sex		Center	
	M	W	M	W	Stat	*p*	Stat	*p*	Stat	*p*	Stat	*p*
Sex, N	431	222	227	99	*χ* ^2^ = 1.299	.25	‐	‐	‐	‐	*χ* ^2^ = 47.31	<10^−6^
Age [years]	34.38 ± 10.38	33.00 ± 10.60	30.43 ± 10.57	29.76 ± 10.15	*F* = 36.17	<.0001	*F* = 3.40	.07	*F* = 0.53	.07	*F* = 71.14	<.0001
Education [years]	14.01 ± 2.36	14.28 ± 2.54	15.47 ± 2.84	15.61 ± 2.88	*F* = 50.62	<.0001	*F* = 3.42	.06	*F* = 0.19	.67	*F* = 9.00	<.0001
Alcohol^a^ [#monthly standard drinks]	207.87 ± 243.36	108.47 ± 108.14	28.66 ± 30.15	17.51 ± 22.05	*F* = 123.53	<.0001	*F* = 13.74	.0002	*F* = 2.87	.09	*F* = 42.10	<.0001
Tobacco^b^ [#monthly cigarettes]	179.11 ± 236.15	144.55 ± 234.93	25.07 ± 91.27	42.85 ± 101.39	*F* = 54.52	<.0001	*F* = 0.55	.46	*F* = 1.10	.30	*F* = 15.37	<.0001

*Note*: For continuous variables, mean ± standard deviation are indicated. Differences in sex distribution across groups and centers were assessed using *χ* ^2^ statistics. Group, sex, group‐by‐sex and center effects on age, education and substance use were evaluated using linear regression models with the following specification: response variable [(1) age, (2) education, (3) alcohol, (4) tobacco] ~ 1 + center + group × sex.

Abbreviations: AUD, alcohol use disorder; *F*, F‐statistics; HC, healthy controls; M, males; *p*, *p*‐value; W, females; *χ* ^2^, *χ* ^2^ statistics.

^a^ Information available for 483 AUD subjects, 211 HC.

^b^ Information available for 460 AUD subjects (*n* = 254 with #monthly cigarettes > 0), 140 HC (*n* = 27 with #monthly cigarettes > 0).

### 3.2 Group, sex, and group‐by‐sex effects on brain volumes (GLM #1)

This section overviews the main effects of group and group‐by‐sex on local GM and WM volumes. The main effects of sex are described in Appendix S1.

#### 3.2.1 AUD group versus control group

**FIGURE 1:**
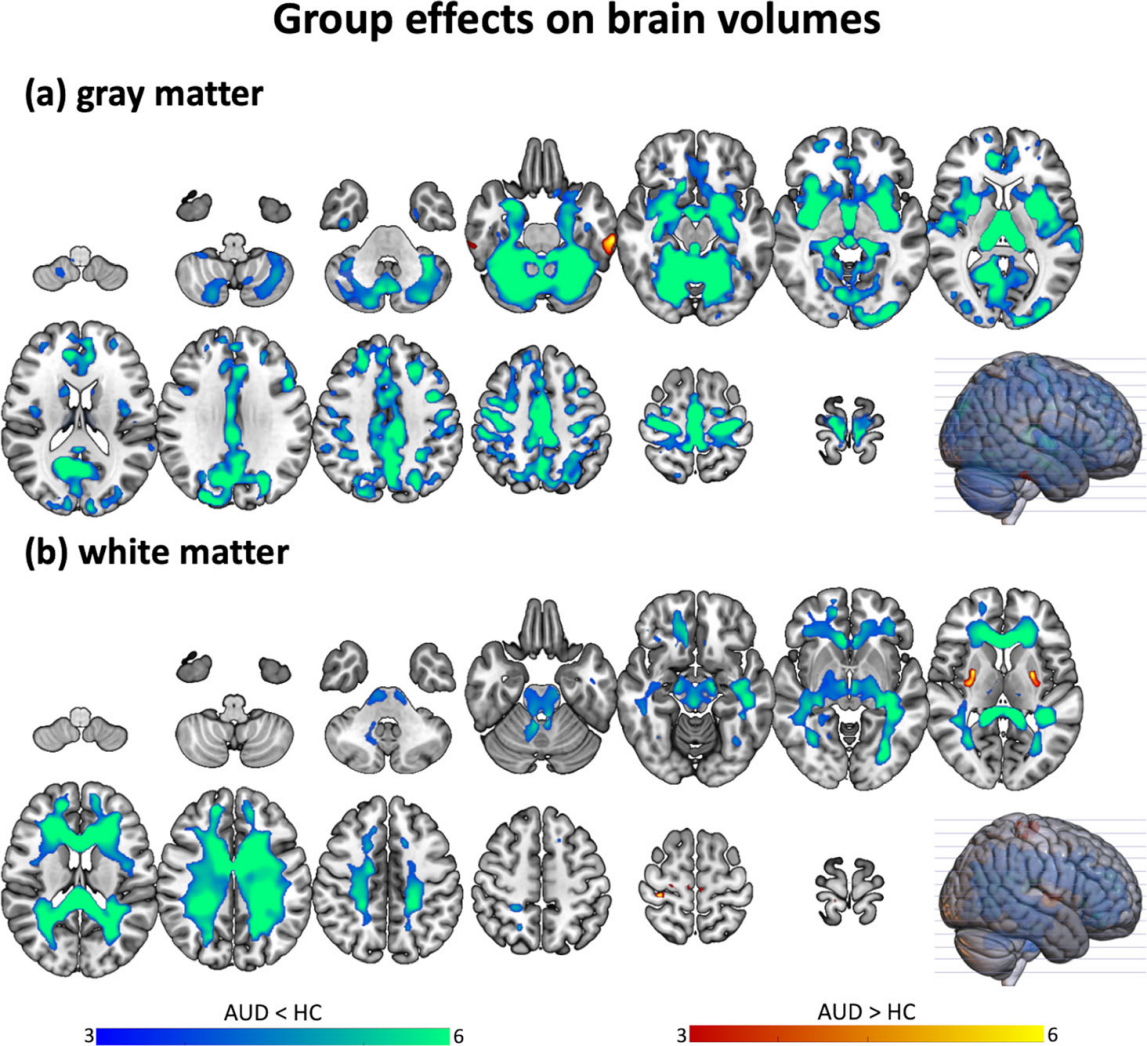
Nonparametric VBM results, main effect of group. Brain clusters showing significant GM volume differences in AUD individuals compared to the control group (*p* < .05, FWE, >50 voxels). All clusters remain significant after threshold‐free cluster enhancement (*p* < .05, FWE). AUD, alcohol use disorder; FWE, peak‐based family‐wise error corrected; GM, gray matter; HC, healthy controls; VBM, voxel‐based morphometry.

Overall, groups showed different GM (Table 2 and Figure 1a) and WM (Table 3 and Figure 1b) volumes in widespread brain areas (*p* < .05, FWE, surviving to TFCE), which are overviewed below.

**TABLE 2:** Nonparametric VBM statistics concerning main effects of AUD, sex, and monthly standard drinks and cigarettes in AUD people on GM volumes.

	Contrast	Atlas region^a^	# voxels	*p* (FWE)	Peak *T*	Peak Cohen's *D*	*x*, *y*, *z* {mm}
Main effect of group (AUD vs. HC)	AUD < HC	Thalamus R	146,167	<.001	14.47	0.93	16, −30, 6
		Orbitofrontal sup R	448	.005	5.31	0.34	28, 57, −4
		Precentral R	149	.009	4.90	0.31	44, 8, 32
		Inferior Temporal L	169	.012	4.72	0.30	39, −15, −28
		Frontal Mid L	112	.014	4.62	0.30	−30, 60, 6
		Cerebellum 9 L	120	.015	4.54	0.29	−8, −44, −54
	AUD > HC	Supramarginal L	333	<.001	6.53	0.42	−57, −33, −30
Main effect of sex (females vs. males)	Females < males	Hippocampus L	28,514	<.001	11.32	0.73	−33, −3, −26
		Inferior Occipital L	2649	<.001	7.74	0.50	−52, −72, −15
		Vermis 3 R	13,702	<.001	7.66	0.49	2, −39, −18
		Sup Temporal L	2154	<.001	7.40	0.48	−68, −34, 12
		Hippocampus R	66	<.001	6.17	0.40	26, −33, 8
		Frontal Sup L	201	.001	6.10	0.39	−12, 69, 15
		Lingual R	161	.001	6.00	0.39	14, −72, 0
		Mid Frontal R	161	.002	5.76	0.37	42, 58, 4
		Mid temporal pole R	86	.007	5.31	0.34	44, 20, −39
		Inf Parietal R	115	.009	5.22	0.34	57, −34, 54
		Cuneus R	81	.017	5.00	0.32	20, −56, 22
Monthly standard drinks	Females < males	Precentral R	85	.001	5.17	0.49	56, −4, 46

*Note*: All statistics are associated with significant threshold‐free cluster enhancement (TFCE) values (*p* < .05, FWE).

Abbreviations: AUD, alcohol use disorder; FWE, peak‐based family wise error correction; GM, gray matter; HC, healthy controls; *p*, *p*‐value; *T*, nonparametric *T*‐statistics.

^a^ The atlas region refers to the peak voxel.

**TABLE 3:** Nonparametric VBM statistics concerning main effects of AUD and sex on WM volumes.

	Contrast	Atlas region^a^	# voxels	*p* (FWE)	Peak *T*	Peak Cohen's *D*	*x*, *y*, *z* {mm}
Main effect of group (AUD vs. HC)	AUD < HC	Cingulum L	74,780	<.001	9.61	0.61	−6, 24, 12
Main effect of sex (females vs. males)	Females < males	Corticospinal R	126,057	<.001	11.15	0.72	8, −8, −14
		Cingulum L	125	<.001	8.30	0.53	−27, −20, −27
		Cingulum R	105	<.001	6.71	0.43	27, −18, −28

*Note*: All statistics are associated with significant threshold‐free cluster enhancement values (*p* < .05, FWE).

Abbreviations: AUD, alcohol use disorder; HC, healthy controls; FWE, peak‐based family wise error correction; *p*, *p*‐value; *T*, nonparametric *T*‐statistics; WM, white matter.

^a^ The atlas region refers to the peak voxel.

##### Gray matter

Regarding GM, as shown in Table 2 and Figure 1, the AUD relative to control group had lower GM volume (small to large effects, ranging from *D* = 0.29 to *D* = 0.93) in the bilateral thalamus, putamen, insula, medial temporal regions (i.e., amygdala, hippocampus, and para‐hippocampal and superior temporal gyri), frontal regions (i.e., cingulate and middle and superior frontal cortices, precentral gyrus), occipital regions (i.e., lingual gyrus, cuneus, calcarine cortex) and vermis and cerebellum. Participants with AUD versus controls showed larger GM volume in the left supramarginal gyrus (small effect size, *D* = 0.42).

##### White matter

The AUD group was characterized by lower WM volume than the control group in a large cluster encompassing the bilateral cingulum and corpus callosum, inferior longitudinal fasciculus, arcuate fasciculus, corticospinal tract, internal capsule, and cerebellar pedunculus (medium effect, *D* = 0.61).

#### 3.2.2 Group‐by‐sex effects

**FIGURE 2:**
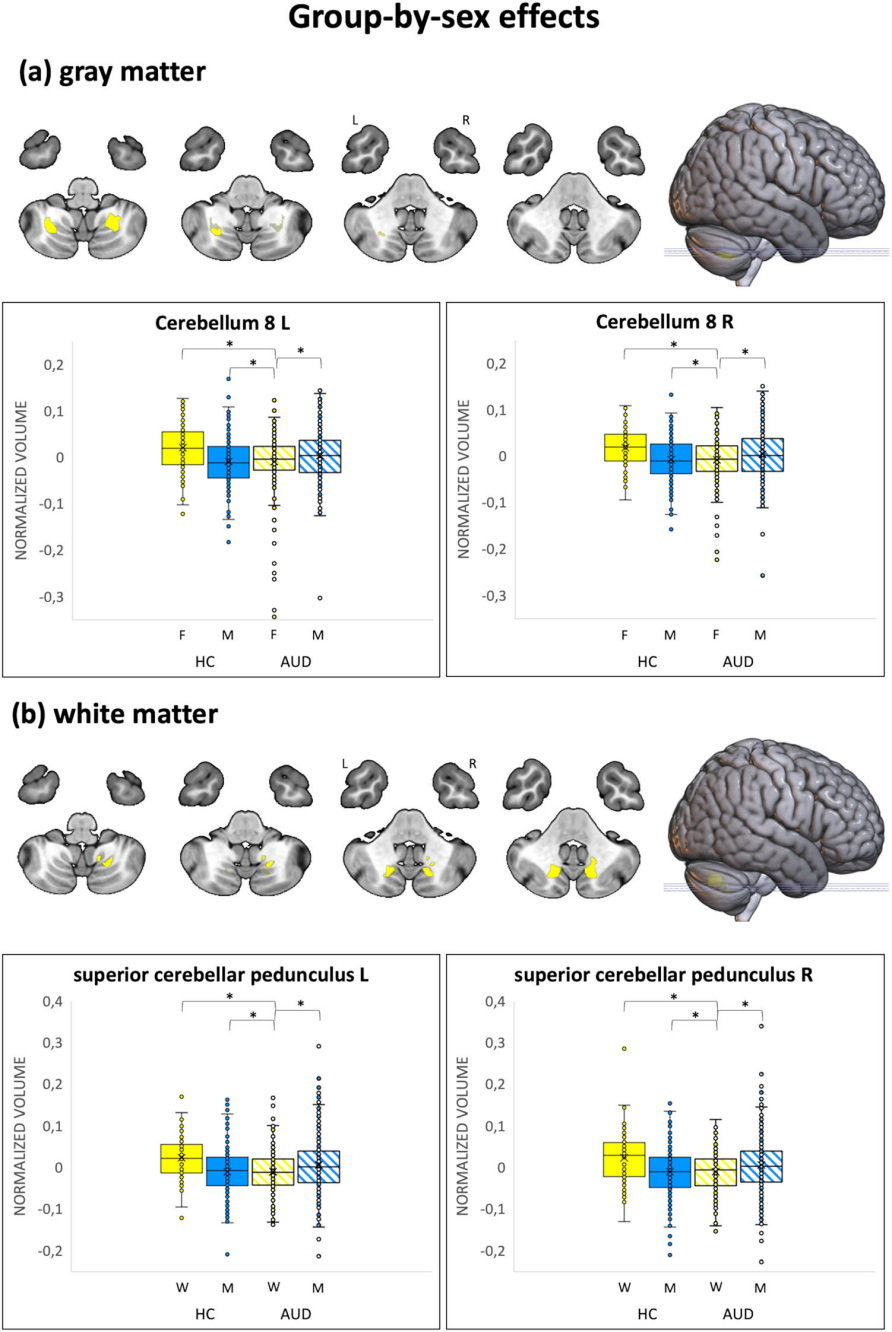
Nonparametric VBM results, group‐by‐sex effects. (A) Brain clusters with significant group‐by‐sex effects on GM volumes (*p* < .05, FWE, >50 voxels). (B) Brain clusters with significant group‐by‐sex effects on WM volumes (*p* < .05, FWE, >50 voxels). All clusters, marked in pink, show greater alcohol‐related volume abnormalities in females relative to males and remain significant after threshold‐free cluster enhancement (*p* < .05, FWE). The box and whisker plots illustrate the volume distribution in the group‐by‐sex subsets in the cluster peaks. The box of the most affected subset is highlighted in bold. Pairwise differences are marked with asterisk symbols (*p < 10^−6^). AUD, alcohol use disorder; FWE, peak‐based family wise error corrected; GM, gray matter; VBM, voxel‐based morphometry; WM, white matter.

Overall, there were significant group‐by‐sex effects on GM (Table 4 and Figure 2a) and WM (Table 5 and Figure 2b) volumes in the brain regions that are overviewed below.

**TABLE 4:** VBM and ROI statistics concerning group‐by‐sex effects on GM volumes.

VBM peak						Cluster ROI analysis	
Atlas region	*p*(FWE)	*p*(unc)	Peak *T*	Peak Cohen's *D*	*x*, *y*, *z* {mm}	# voxels	Pairwise differences
Cerebellum 8 L	.001^a^	<.001	5.52	0.35	−21, −56, −46	127	AUD F < HC F** (*D* = 0.40) AUD F < AUD M** (*D* = 0.45) AUD F < HC M** (*D* = 0.38)
Cerebellum 8 R	.002^a^	<.001	5.34	0.34	27, −62, −45	89	AUD F < HC F** (*D* = 0.38) AUD F < AUD M** (*D* = 0.49) AUD F < HC M** (*D* = 0.40)
Cerebellum crus 1 L	n.s.	<.001	3.50	0.22	−27, −60, −34	83	AUD F < HC F** (*D* = 0.39) AUD F < AUD M** (*D* = 0.45) AUD F < HC M** (*D* = 0.57)
Cerebellum 6 R	n.s.	<.001	3.39	0.22	28, −58, −33	90	AUD F < HC F** (*D* = 0.39) AUD F < AUD M** (*D* = 0.37) AUD F < HC M** (*D* = 0.50)
Mid cingulum R	n.s.	<.001	4.13	0.27	8, −27, 45	267	AUD F < HC M** (*D* = 0.53) AUD M < HC M** (*D* = 0.50) HC F < HC M** (*D* = 0.38)
Superior temporal pole L	n.s.	<.001	3.95	0.25	−16, 10, −27	78	AUD M < HC M** (*D* = 0.36)
Mid occipital R	n.s.	<.001	3.86	0.25	34, −93, 15	153	AUD F < HC M* (*D* = 0.26) AUD M < HC M** (*D* = 0.29) HC F < HC M** (*D* = 0.32)

Abbreviations: AUD, alcohol use disorder; *D*: Cohen's *D* effect size; F, females; GM, gray matter; HC, healthy subjects; M, men; *p*(FWE), permutation‐based *p*‐value after peak‐based family wise error correction; *p*(unc): uncorrected permutation‐based *p*‐value; ROI, region of interest; *T*, *T*‐statistics; VBM, voxel‐based morphometry.

^a^ Statistics associated with significant threshold‐free cluster enhancement values (*p* < .05, FWE).

* *p* < .001.

** *p* < 10^−6^.

**TABLE 5:** VBM and ROI statistics concerning group‐by‐sex effects on WM volumes.

VBM peak						Cluster ROI analysis	
Atlas region	*p*(FWE)	*p*(unc)	Peak *T*	Peak Cohen's *D*	*x*, *y*, *z* {mm}	# voxels	Pairwise differences
Superior cerebellar pedunculus L	<.001	<.001	5.83	0.37	−9, −74, −36	347	AUD F < HC F** (*D* = 0.32) AUD F < AUD M** (*D* = 0.50) AUD F < HC M** (*D* = 0.41)
Superior cerebellar pedunculus R	<.001	<.001	5.58	0.36	15, −74, −36	251	AUD F < HC F** (*D* = 0.37) AUD F < AUD M** (*D* = 0.51) AUD F < HC M** (*D* = 0.49)
Cingulum L	n.s.	<.001	3.85	0.25	−16, 40, −10	891	AUD F < HC F* (*D* = 0.28) AUD F < AUD M** (*D* = 0.57) AUD F < HC M** (*D* = 0.50)
Anterior commissure L	n.s.	<.001	3.95	0.25	−15, 4, −6	398	AUD F < HC F* (*D* = 0.26) AUD F < AUD M** (*D* = 0.67) AUD F < HC M** (*D* = 0.58) HC F < AUD M* (*D* = 0.22)
Uncinate R	n.s.	<.001	3.66	0.23	36, −6, −16	343	AUD F < AUD M** (*D* = 0.53) AUD F < HC M** (*D* = 0.40)
Uncinate L	n.s.	<.001	3.51	0.23	−32, −6, −14	59	AUD F < HC F* (*D* = 0.27) AUD F < AUD M** (*D* = 0.51) AUD F < HC M** (*D* = 0.48)

*Note*: All statistics are associated with significant threshold‐free cluster enhancement values (*p* < .05, FWE).

Abbreviations: AUD, alcohol use disorder; *D*, Cohen's *D* effect size; F, females; HC, healthy subjects; M, men; *p*(FWE), permutation‐based *p*‐value after peak‐based family wise error correction; *p*(unc): uncorrected permutation‐based *p*‐value; ROI, region of interest; *T*, *T*‐statistics; VBM, voxel‐based morphometry; WM, white matter.

* *p* < .001.

** *p* < 10^−6^.

##### Gray matter

As shown in Table 4 and Figure 2, group‐by‐sex effects on voxel‐based GM volumes were observed in the bilateral cerebellum (portion 8) (*p* < .05, FWE, surviving to TFCE). Trend‐level group‐by‐sex effects (*p* < .001) were also observed in (i) the left cerebellum crus 1 and superior temporal pole, (ii) the right cerebellum 6, middle cingulate cortex, and middle occipital cortex. Post‐hoc ROI analyses showed that the cerebellar clusters had lower GM volume in AUD females versus all other groups (small to medium effects, ranging from *D* = 0.37 to *D* = 0.57). Conversely, lower GM volume in AUD versus control males but not in AUD versus control females was found in (i) the left superior temporal pole, (ii) the right middle occipital and middle cingulate cortices (small to medium effects, ranging from *D* = 0.29 to *D* = 0.50).

##### White matter

As shown in Table 5 and Figure 2b, group‐by‐sex effects on WM volumes were observed in the bilateral superior cerebellar pedunculus, in clusters adjacent to the GM cerebellar clusters (*p* < .05, FWE, surviving to TFCE).

At the trend level, group‐by‐sex interaction effects were observed in (i) the bilateral uncinate, (ii) the left cingulum and anterior commissure. Post‐hoc ROI analyses showed that WM volume in these tracts was lower in AUD females than in males and, with the exception of the right uncinate, than in female controls (small to medium effects, ranging from *D* = 0.27 to *D* = 0.67).

### 3.3 Effects of monthly standard drinks and cigarettes on brain volumes in AUD (GLM #2)

This section overviews the main effects of monthly standard drinks and monthly cigarettes on GM and WM volumes in AUD females and/or males, and females relative to males.

**FIGURE 3:**
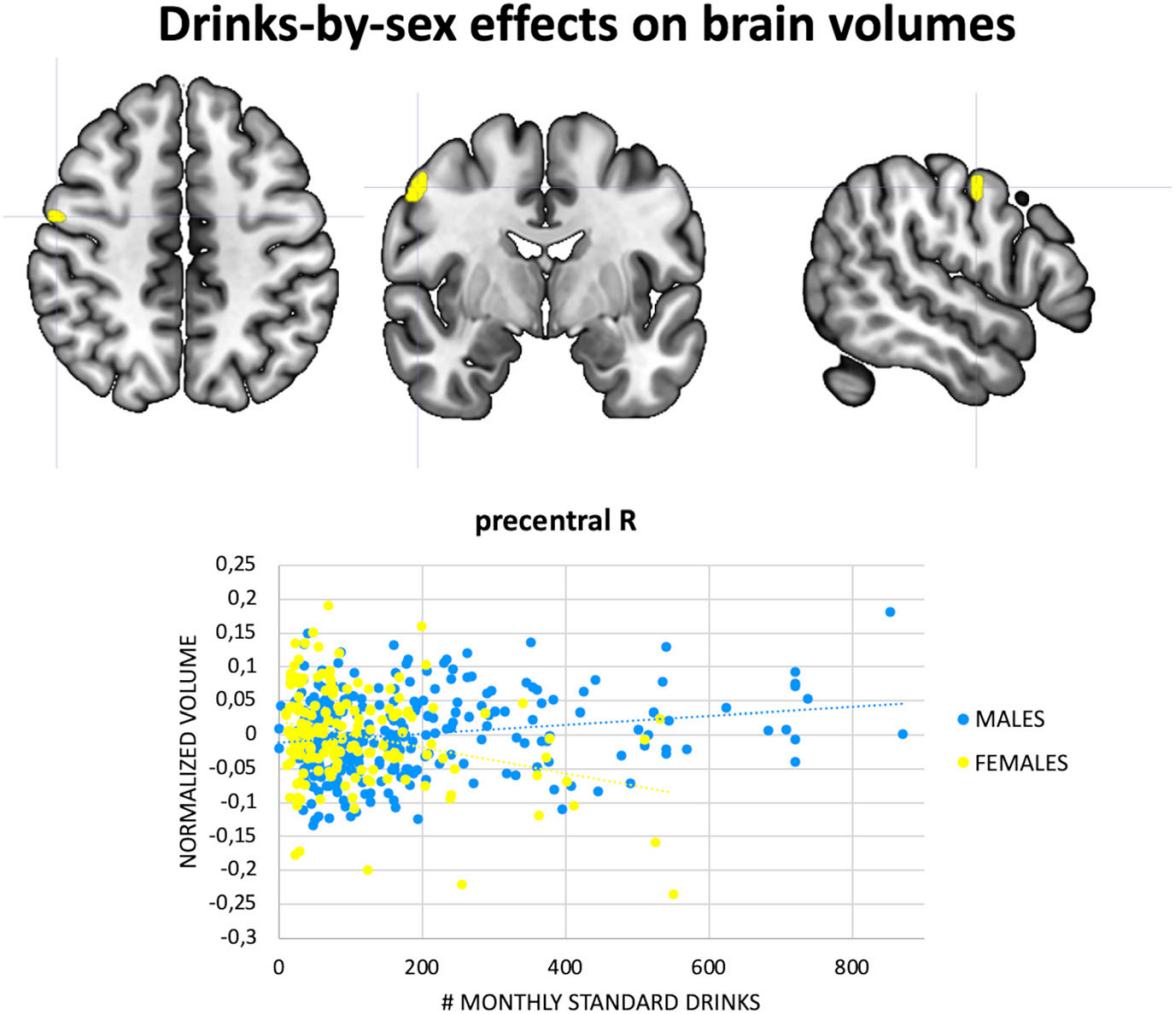
VBM results, alcohol use‐by‐sex effects. GM cluster with significant interaction effects between monthly standard drinks and sex in AUD subjects (*p* < .05, FWE, >50 voxels). The cluster showed greater alcohol use‐related volume reduction in females relative to males and remain significant after threshold free cluster enhancement (*p* < .05, FWE). The scatterplots illustrate the association between GM volume in the cluster peak and monthly standard alcohol drinks in females (pink dots) and males (green dots), together with the relative linear interpolations.

An interaction effect between monthly standard drinks and sex on GM volume was observed in a portion of the right precentral gyrus (*p* < .05, FWE, surviving to TFCE). Specifically, higher monthly standard alcohol drinks were associated with lower precentral GM volume in females with AUD, whereas a weaker but opposite association was found in males with AUD (Table 2, Figure 3). The effect size was medium (*D* = 0.49). There were no significant effects of monthly standard drinks on GM in females, males, shared between sexes, as well as no effects of monthly standard drinks on WM in females, males, shared between sexes, or in interaction with sex. Likewise, there were no other effects of monthly cigarettes on GM and WM volumes in females, males, females and males examined together, or in interaction with sex (*p* < .05, FWE).

## 4 DISCUSSION

In our study, using an unbiased whole‐brain local volumetry approach, we found widespread effects of AUD on GM and WM volumes, partly shared and partly different between sexes.

We replicated prior findings that AUD was associated with lower GM and WM volumes in striatal, thalamic, cerebellar, and frontotemporal brain networks. We also found group‐by‐sex effects on cerebellar GM and WM regions, which were more affected by AUD in females than in males. Trend‐level group‐by‐sex effects were observed in frontotemporal WM regions, in the same direction as cerebellar ones, as well as in GM regions in the reward and executive networks (incl. middle cingulate cortex and superior temporal lobe)—which were more affected by AUD in males than in females. In females with AUD, greater monthly standard drinks were associated with lower GM volume in a portion of the right precentral gyrus, whereas an opposite (though weaker) association was found in males with AUD.

This evidence underscores the importance of examining both sex‐common and sex‐specific neurobiological mechanisms associated with AUD, supporting the hypothesis that sex‐specific risk factors and consequences of AUD might be associated with partially distinct WM and GM volume alterations between females and males. In this line, sex differences in alcohol‐related cerebellar and reward and executive network impairments might underlie the different relationships between alcohol use and affective and cognitive processes between females and males.

Within the context of the ENIGMA Addiction initiative, we extended previous evidence of alcohol‐by‐sex effects on selected brain regions by employing a whole‐brain perspective. Thus, our study differed from the manuscripts from Rossetti et al., and Grace et al., almost exclusively in terms of methodology. In this respect, our findings rely on a volume‐based analysis that substantially differs from the surface‐based analysis employed in our previous studies, opening doors to future methodological comparisons.

### 4.1 AUD effects on widespread subcortical, cortical, and cerebellar regions

Compared to controls, individuals with AUD had lower GM volume in large portions of the bilateral cerebellum, thalamus, putamen, amygdala, hippocampus as well as frontal, temporal, and occipital regions, revealing a complex picture of differences targeting brain areas involved in motivational, disinhibitory, executive, and cognitive processes.

Our findings confirm prior knowledge of alcohol‐related volume decreases in key nodes and connections of the striatal, thalamic, and cerebellar circuits, which however was obtained from small samples (Pitel et al., 2012, 2015; Sullivan et al., 2005; Zahr et al., 2010). Moreover, the putamen exerts a key role in control of habitual behaviors, including chronic alcohol use (Tricomi et al., 2009), and showed alcohol‐related alterations in volume (Sullivan et al., 2005), dopaminergic metabolism (Volkow et al., 2017), alcohol‐cue reactivity (Sjoerds et al., 2014), interactions with the prefrontal cortex (Courtney et al., 2013), and synaptic susceptibility (Cuzon Carlson et al., 2011). In the thalamus, lower alcohol‐related volume (Pitel et al., 2012, 2015), hyperactivation in response to alcohol cues and conflict anticipation (George et al., 2001; Hu et al., 2015), and functional circuitry differences (Zhornitsky et al., 2018) have been observed. As regards the cerebellum, genes associated with familial risk for AUD were proposed to influence its development (Hill et al., 2011), suggesting that this region might be involved in the etiology of AUD. Lower cerebellar volume, mitochondrial dysfunction, and lower long‐range functional connections might especially contribute to the behavioral, cognitive, and motor alterations of AUD (Chen et al., 2019; Jung, 2015; Zahr et al., 2010).

Likely, our findings provide strong support to the abnormalities in prefrontal and insular regions that have been commonly associated with AUDs (Xiao et al., 2015), and to the volume reduction in the limbic system in AUD compared to control subjects, which confirms evidence from both preclinical (Morris et al., 2010) and clinical studies on independent AUD samples (Dager et al., 2015; Suzuki et al., 2010). Of note, Dager and colleagues hypothesized an involvement of the amygdala in the genetic predisposition to AUD, paving the way to future investigations on its possible role of mediator between genetics and illness onset.

The widespread WM volume reduction observed in our AUD sample is reflected to a large extent in a recent meta‐analytic evidence regarding VBM studies on AUDs (Monnig et al., 2013). Notably, our results of lower WM volume in cingulum, corpus callosum, and internal capsule have been found among the significant clusters with both macro‐ and micro‐structural WM alterations in AUD in an integrated VBM and DTI meta‐analysis (Spindler et al., 2022).

### 4.2 Corticocerebellar abnormalities in females with AUD

Among the GM regions and WM tracts differentially affected by AUD between sexes, those in the cerebellum showed more pronounced alcohol‐related volume abnormalities in females. The size of this interaction effect (in the small to medium range) was most pronounced in the WM connections, in support of the hypothesis that females might be more vulnerable to alcohol‐related WM deficits than males (Mann et al., 2005; Pfefferbaum et al., 2009). Furthermore, alterations in frontotemporal WM tracts were more pronounced in females with AUD, even when the effect size of the alteration was small.

Cerebellar and frontotemporal network alterations have been associated with AUD (Rogers et al., 2012; Sullivan et al., 2003) and familial history of alcoholism (Herting et al., 2011; Tessner & Hill, 2010), suggesting their role as potential risk biomarkers for AUD. Of note, the group‐by‐sex effects on cerebellar GM volume confirm the robustness of the recent findings from our group (Rossetti et al., 2021), which were obtained on a largely overlapping sample examined using a surface‐based analysis distinct from the whole brain, local, volume‐based analysis of the current study. In independent samples, apart from the greater long‐term effects of heavy drinking on anterior cerebellar volumes in males than in females (Sawyer et al., 2016), to our knowledge there is no other literature evidence of alcohol‐by‐sex effects on cerebellar and frontotemporal circuits. As already mentioned, this limited evidence is partly due the paucity and limited statistical power of neuroimaging studies examining sex differences in AUD (Pfefferbaum & Sullivan, 2002).

Our finding might reflect the observed sexual dimorphisms in cerebellar and frontotemporal circuit volumes from development onwards (Delvecchio et al., 2021; Ritchie et al., 2018; Sullivan et al., 2020). Furthermore, due to their well‐known frontal and cerebellar involvement in cognitive processing, alcohol‐by‐sex effects on corticocerebellar networks might underlie the earlier appearance of cognitive impairment in females versus males, that is sometimes observed in the course of AUD (Fama et al., 2020). The enhanced reduction of the uncinate and cingulum bundle volumes characterizing AUD females compared to males suggests sex‐specific correlates of AUD that might contribute to differences in affective and interoceptive behaviors (Oishi et al., 2015). Since abnormalities in such behavioral features and in the functional connectivity of the underlying brain networks are characteristic of depression (Bondi et al., 2023; Guo et al., 2013; Riva‐Posse et al., 2014), the observed alterations might explain the more frequent comorbid depression in alcohol dependent females than males, supporting our preliminary hypotheses. Lastly, in females but not males with AUD there was a negative association between monthly standard drinks and GM volume in the right precentral gyrus. This interaction effect is supported by recent evidence of a positive association between right precentral volume and alcohol use in males (Luciana et al., 2013).

### 4.3 Reward and executive network abnormalities in males with AUD

In our AUD sample, males but not females were characterized by lower GM volume than same‐sex controls in nodes of the central executive, sensorimotor, and salience networks, particularly in middle cingulate, superior temporal, and middle occipital regions. The effect size for this volume reduction in males with AUD was in the small to medium range, whereas the relative group‐by‐sex effects were of small size.

Previous findings on AUDs have shown volumetric alterations in brain regions that play a role in reward processing (Makris et al., 2008). Furthermore, smaller reward system volumes in males with AUD compared to both control males and females with AUD have been described in the literature (Verplaetse et al., 2021).

We were the first to report group‐by‐sex effects on temporal, occipital and cingulate regions, but neuroimaging studies on males with AUD partly support our findings. Indeed, the smaller volume in the superior temporal region might be associated with the alterations of glutamate metabolism that have been recently found in a PET study on abstinent AUD males. Our occipital finding is in line with fMRI evidence that the middle occipital gyrus is more activated by alcohol cues in males with AUD versus control males (Wrase et al., 2007). Reduced functional connectivity of the cingulum was observed during imagery of alcohol cues in a mostly male sample (>70%) of individuals with AUD relative to controls (Zakiniaeiz et al., 2017). Moreover, lower resting‐state connectivity within the salience and reward networks—including middle cingulate cortex and middle occipital gyrus as nodes—was found in abstinent males with AUD compared to control males and females (Muller‐Oehring et al., 2015). Finally, the absence of group‐by‐sex effects on limbic regions that we recently observed in an almost overlapping sample (Grace et al., 2021; Rossetti et al., 2021) might be due to methodological differences between our study and previous ones, regarding both extraction of brain morphological features and statistical inference tools.

### 4.4 Study limitations and future perspectives

The present study has some limitations. The integration of datasets previously collected from multiple cohorts in the ENIGMA Addiction working group centers required us to address nuisance and confounding effects, but at the same time has made the findings more representative of the worldwide population. Accordingly, the inconsistent inclusion criteria and diagnostic instruments across centers increased the heterogeneity of the sample in terms of substance use, but also the generalizability of the obtained results concerning AUD. Information on chronicity of AUD, days of abstinence from alcohol, severity of alcohol use, or concurrent drug use was not uniformly collected across centers, impeding the exploration and control of their effects on brain anatomy.

Although the imbalance in (i) the size of AUD and control groups, (ii) the number of females and males per group might have influenced the results, the similar proportion of females and males in the two groups reinforces the reliability of the detected group‐by‐sex effects. Our findings are limited by the fact that we did not conduct chromosomal genetic testing, anatomical inspection, or hormonal assessment to verify the biological sex. Another limitation to consider is the unequal distribution of AUD and control individuals across centers, which however was taken into account in the VBM design.

Future comparisons on more balanced, independent datasets are needed to confirm our results. In this regard, the impact of monthly standard drinks on cerebellar volume in males, which was detected using a cluster‐based FWE correction, should be replicated on bigger samples using the more stringent peak‐based FWE correction. These results should be integrated with longitudinal analyses, which can provide evidence of similarities and differences between males and females in the progressive effects of AUD on neuroanatomy and brain aging. Future research works might specifically address any exacerbation of age‐related changes induced by alcohol use, as well as any similarity between age‐ versus alcohol‐related volume changes as a function of alcohol use severity.

Lastly, the complementary methodological approaches between our and previous ENIGMA Addiction works on alcohol‐by‐sex effects on brain anatomy pave the way to robust methodological comparisons between (i) surface‐based and volume‐based, (ii) region‐based and voxel‐based approaches applied to our large dataset. Quantitative comparisons between different types of brain morphological features will be the subject of upcoming investigations.

## 5 CONCLUSIONS

In the present study, using a whole‐brain exploratory approach, we showed that the local GM and WM volume characteristics of AUD are partially shared but also partially distinct between groups of females and males. This evidence underlines the need to consider the sexually dimorphic biological mechanisms involved in AUD, to develop more accurate patient‐centered addiction models and in turn more effective intervention strategies.

## AUTHOR CONTRIBUTIONS

Eleonora Maggioni: Contributed new analytical tools; Analyzed data; Wrote the paper. Maria G. Rossetti: Analyzed data; Methodology review; Writing – review & editing. Nicholas B. Allen, Albert Batalla, Marcella Bellani, Yann Chye, Janna Cousijn, Anna E. Goudriaan, Robert Hester, Kent Hutchison, Chiang‐Shan R. Li, Rocio Martin‐Santos, Reza Momenan, Rajita Sinha, Lianne Schmaal, Nadia Solowij, Chao Suo, Ruth J. van Holst, Dick J. Veltman, Murat Yücel: Performed research; Writing – review & editing. Patricia Conrod, Scott Mackey, Hugh Garavan: Designed research; Performed research; Writing – review & editing. Paul M. Thompson: Designed research; Project administration; Writing – review & editing. Paolo Brambilla: Performed research; Supervision; Methodology Review; Writing – review & editing. Valentina Lorenzetti: Designed research; Performed research; Methodology review; Writing – review & editing.

## CONFLICT OF INTEREST STATEMENT

The authors declare no conflicts of interest.

## Supporting information


**Appendix S1:** Supporting information.

## Data Availability

The clinical and MRI datasets supporting the current study have not been deposited in a public repository because of privacy and ethical restrictions, but are available from the corresponding author on request. The Matlab analysis codes are available at the Open Science Framework web application link https://osf.io/detwu/.
