# Supplementary material for: Brain volumes in alcohol use disorder: Do females and males differ? A whole‐brain magnetic resonance imaging mega‐analysis
Source: Hum Brain Mapp. 2023 Jul 12;44(13):4652–66. doi: 10.1002/hbm.26404 (PMC10400785; doi:10.1002/hbm.26404)
Supplement: Supplementary file 1 — Appendix S1: Supporting information. [file HBM-44-4652-s001.docx]

**Supplementary Material for**

Brain volumes in alcohol use disorder: do females and males differ? A whole-brain Magnetic Resonance Imaging mega-analysis.

Eleonora Maggioni, Maria G. Rossetti, Nicholas B. Allen, Albert Batalla, Marcella Bellani, Yann Chye, Janna Cousijn, Anna E. Goudriaan, Robert Hester, Kent Hutchison, Chiang-Shan R. Li, Rocio Martin-Santos, Reza Momenan, Rajita Sinha, Lianne Schmaal, Nadia Solowij, Chao Suo, Ruth J. van Holst, Dick Veltman, Murat Yücel, Paul M. Thompson, Patricia Conrod, Scott Mackey, Hugh Garavan, Paolo Brambilla^^^, Valentina Lorenzetti^^^.

Corresponding author: Paolo Brambilla, [paolo.brambilla1@unimi.it](mailto:paolo.brambilla1@unimi.it)

**The document includes:**

Supplementary methods

Supplementary results

Fig. S1

Tab. S1

Supplementary references.

**Supplementary methods.**

Center information

For each site within the ENIGMA Addiction Working Group, the details on sample numerosity and clinical characteristics, inclusion and exclusion criteria, substance use measures, MRI scanner, and T1-weighted sequence parameters are reported in Tab. S1.

Sample selection

The original sample (n=1348) was screened using the following exclusion criteria: in the alcohol group, (i) lifetime and/or current primary psychiatric disorders other than alcohol dependence and/or current dependence on substances different from alcohol (n=191), (ii) current abstinence from alcohol for more than 30 days (n=15); in both groups, (i) intellectual quotient < 80 (n=15), (ii) missing data for key variables including sex (n=68) and years of education (n=72), (iii) poor image or brain tissue segmentation quality (n=8). This selection phase resulted in a dataset composed of 979 participants.

General Linear Model analyses

*GLM design specification and estimation*

GLM analysis #1 examined the impact of group (alcohol use disorder (AUD), healthy control (HC)), sex (females, males), and group-by-sex on local GM and WM volumes in the entire dataset (n=979 subjects, of which n=326 control and n=653 with AUD). Voxel-wise GM or WM volumes were modeled in terms of group, sex and age (both interacting with group). Years of education and total intracranial volume (TIV, previously harmonized across centers using ComBat) were included in the model as covariates.

GLM analysis #2 examined the impact of monthly standard drinks and monthly cigarettes on local GM and WM volumes in the AUD group. The GLM design included AUD people for whom information on number of monthly standard drinks and cigarettes was available (n=453, of which n=285 males and n=168 females). Voxel-wise GM or WM volumes were modeled in terms of sex, number of monthly standard drinks (square root transformed), number of monthly cigarettes (square root transformed) and age (all interacting with sex). Years of education and harmonized TIV were included in the model as covariates.

In all analyses, the GLM design specification was followed by the voxel-level estimation of the GLM coefficients via restricted maximum likelihood (REML) algorithm.

*Voxel-based morphometry (VBM) analyses*

The VBM statistical comparisons were performed by considering the only voxels within the group-level optimal threshold GM and WM masks computed from the pre-processed images using the SPM12 Masking toolbox.

Inference was based on nonparametric permutation tests on the GLM *t-*contrasts using the Smith permutation method (n=5000, weighting extent parameter E=0.5, H=2).

Specifically, in GLM analysis #1, the GM or WM differences between subjects with AUD and controls were identified using permutations tests on the *t*-contrasts comparing the AUD and HC regressors; the GM or WM differences between females and males common to AUD and control subjects were extracted via permutations on the *t-*contrast selecting both group-specific sex regressors; group-by-sex interaction effects were assessed via permutations tests on the difference between the group-specific sex regressors.

In GLM analysis #2, the effects of alcohol and nicotine use severity on GM or WM volumes in AUD females and males separately were assessed via permutations on the sex-specific substance use GLM coefficients; the effects common to AUD females and males were assessed via permutations on the *t*-contrast selecting the two sex-specific substance use regressors; permutation tests on the difference between these coefficients allowed to examine any interaction effects between substance use severity and sex on GM or WM volumes.

In all GLM analyses, the significance threshold was set to p=0.05 after peak-based Family Wise Error (FWE) correction. Additional nonparametric analyses using threshold-free cluster enhancement (TFCE) technique (p< 0.05, FWE corrected) were performed to mark the most significant clusters from the main analysis, i.e., those that survived also the TFCE. In GLM analysis #1, group-by-sex effects were also assessed at a trend level (p<0.001). An arbitrary cluster-based correction was performed (minimum cluster size of 50 voxels).

The neuroanatomical location of the significant clusters was defined using the Automated Anatomical Labeling (AAL) atlas for GM analyses (Tzourio-Mazoyer et al., 2002), the NatBrainLab tractography-based atlas for WM analyses (Catani & Thiebaut de Schotten, 2008). Clusters outside the reference atlas were not considered. For all significant clusters, Cohen’s D effect sizes were computed from the T values and degrees of freedom (df) (D=2*T/√df).

*Post-hoc Region-of-interest (ROI) analyses*

Post-hoc analyses were performed on the GM or WM clusters resulting from the VBM group-by-sex contrasts in GLM #1 (p<0.05, FWE, ≥ 50 voxels) using in-house Matlab scripts. The GM or WM volume of each ROI was computed as the sum of GM or WM density values of the voxels within it. In separate GLM designs, using the *fitglm* Matlab function, the resulting ROI volumes were modeled as a function of group-by-sex subset (1. control females, 2. control males, 3. AUD females, AUD males), age, years of education, and TIV. After estimation of the GLM coefficients through least-squares fitting, we made inference on the pairwise differences between group-by-sex subsets through two-sided *t*-tests on the corresponding GLM coefficients (i.e., 1. females with AUD vs. female controls, 2. females with AUD vs. males with AUD, 3. males with AUD vs. male controls, 4. males with AUD vs. male controls, 5. males with AUD vs. female controls, 6. female controls vs. male controls). Significance was set to p=0.001 (multiple comparisons were already accounted for in the GLM design). For all significant clusters, Cohen’s D effect sizes were computed from the T values and df, as specified above.

**Supplementary results**

Comparison between females and males

The brain clusters with voxel-based GM or WM volume differences between females and males are illustrated in Fig. S1 (p<0.05, FWE). Compared to males, females were characterized by lower GM volume in clusters located in (i) the bilateral vermis and cerebellum (lobules 3, 4-5, and 6), basal ganglia (i.e., putamen, pallidum, caudate), frontal (i.e., inferior frontal cortex), cingulate (middle and posterior cingulate cortex), and temporal (i.e., hippocampus, para-hippocampal gyrus, amygdala, insula, and fusiform and temporal gyri) lobes, and in (ii) the left superior frontal cortex. The effect sizes were small to medium (D=0.35-0.60 range). Females also showed lower volume in WM tracts located in proximity of the temporal and frontal GM clusters, involving the bilateral arcuate, inferior longitudinal and occipitofrontal fasciculi, corticospinal tract, cingulum and corpus callosum, internal capsule, cortico-ponto-cerebellar tract, and inferior cerebellar pedunculus. The effects were of medium size (D=0.43-0.72 range). There were no brain regions with lower GM or WM volume in males compared to females.

# Supplementary figures and tables


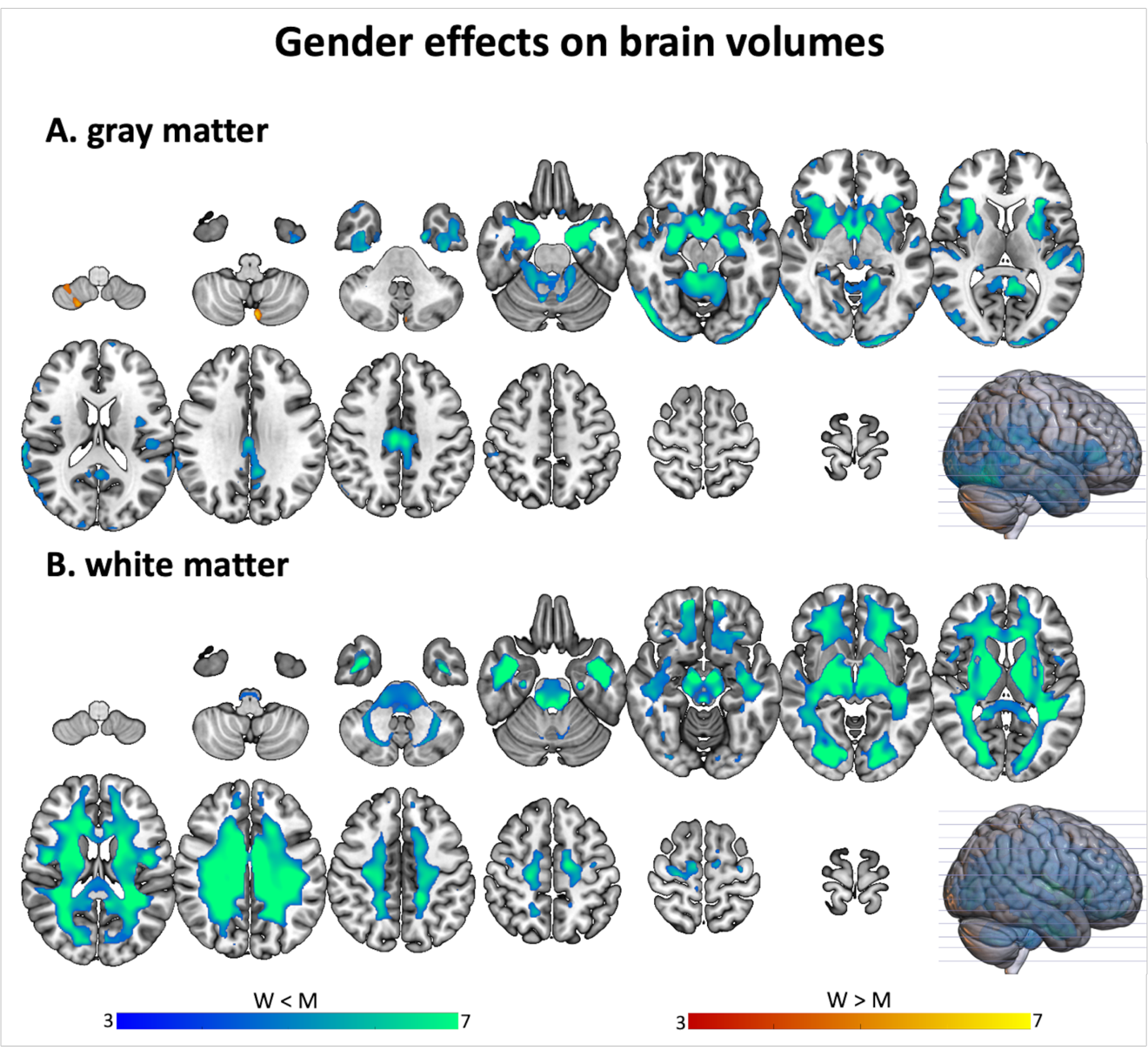


**Fig S1. Nonparametric VBM results, main effect of sex.** A panel. Brain clusters showing significant GM volume differences in females compared to males (p<0.05, FWE, > 50 voxels). B panel. Brain clusters showing significant WM volume reduction in females compared to males (p<0.05, FWE, > 50 voxels). VBM: voxel-based morphometry. GM: gray matter. WM: white matter. FWE: peak-based family wise error corrected.

**Tab. S1. Sample and assessment details per imaging site.**

|  | **Site 1** | **Site 2** | **Site 3** | **Site 4** | **Site 5** | **Site 6** | **Site 7** | **Site 8** | **Site 9** | **Site 10** |
| --- | --- | --- | --- | --- | --- | --- | --- | --- | --- | --- |
| *N_tot_* | 442 | 186 | 129 | 352 | 62 | 52 | 40 | 29 | 18 | 38 |
| *N_included_ (AUD, HC)* | 361 (361, 0) | 154 (154, 0) | 116 (31, 85) | 134 (55, 79) | 61 (41, 20) | 35 (11, 24) | 40 (0, 40) | 25 (0, 25) | 18 (0, 18) | 35 (0, 35) |
| PI | Hutchison | Hutchison | Sinha | Momenan (Grodin, Lin, Durkee, Hommer, & Momenan, 2013; Momenan et al., 2012; Senatorov et al., 2015) | Sjoerds/  Veltman | Goudriaan/Van Holst  (van Holst, Clark, Veltman, van den Brink, & Goudriaan, 2014) | Cousijn/  Goudriaan | Martin-Santos | Solowij | Yucel |
| Study name | ETOH | Olanzapine | IRC | NIAAA | NESDA-AD | ADPG study | Cannabis Prospective | Chronic cannabis users (Barcelona) | Chronic Cannabis | Chronic Cannabis - Memory |
| **Location** |  |  |  |  |  |  |  |  |  |  |
| **Inclusion criteria**  **(AUD)** | Alcohol dependence (DSM-IV; APA, 1994) | ≥ 5 drinks/ per occasion (males)  ≥ 4 drinks/ per occasion (females)  ≥ 5 times in the past month //desire to quit drinking | Alcohol dependence (DSM-IV; APA, 1994) | Alcohol dependence  (DSM-IV; APA, 1994) | Alcohol dependence (DSM-IV; APA, 1994) | Alcohol abuse or dependence (DSM-IV; APA, 1994),  alcohol abstinence >/= 2 weeks | - | - | - | - |
| **Exclusion criteria (all participants)** | | | | | | | | | | |
| Axis I psychiatric disorders | SCID | SCID | SCID | SCID | MINI plus | CIDI | MINI | PRISM, medical history | SCID | SCID, medical history |
| Urine toxicology (MRI day) | Positive screens | Positive screens | Positive screens | Positive screens | Positive screens | Positive screens | Positive screens (alcohol, illicit) | Positive screens (alcohol, illicit) | Positive screens (alcohol, illicit) | Positive screens (alcohol, illicit) |
| Breath test  (MRI day) | Positive | Positive | Positive | Positive | Positive | - | - | - | - | - |
| General MRI contraindication | yes | yes | yes | yes | yes | yes | yes | yes | yes | yes |
| Other substances, Current abuse or dependence | yes | yes | yes | no | yes | yes | yes | yes | yes | no |
| Use of psychoactive medication | yes | no | no | no | no | no | yes | yes | yes | yes |
| Others | Severe alcohol withdrawal | Severe alcohol withdrawal |  | IQ < 80 | - Use of psychoactive medication | age < 18 years, IQ < 80 |  | Left-handedness, female | Left-handedness |  |
| **MRI sequence parameters** | | | | | | | | | | |
| Voxel size, mm^3^ | 1×1×1 | 1x1×1 | 1×1×1 | 0.9×0.9×1-1.5 | 1×1×1 | 1×1×1 | 1×1×1.2 | 1.17×1.17×1.2 | 1×1×1.1 | 1×1×1.1 |
| MR sequence | MPRAGE | MPRAGE | MPRAGE | MPRAGE | Gradient Echo | Gradient Echo | Turbo Field Echo | Fast Spoiled Gradient Inversion-Recovery | Spoiled Gradient Recalled Echo | MPRAGE |
| Echo | 5-echo multi-echo | 5-echo multi-echo |  |  |  |  |  |  |  |  |
| TR, ms | 2350 | 2350 | 2350 | 4.5-7.8 | 9 | 9 | 9.6 | 11.8 | 6.4 | 1900 |
| TE, ms | 1.64, 3.5, 5.36, 7.22 & 9.08 | 1.64, 3.5, 5.36, 7.22 & 9.08 | 3.34, 1 | 2.2-3.1 | 3.6 | 3.6 | 4.6 | 4.2 | 2.9 | 2.15 |
| Flip angle | 7° | 7° | 7° | 6° | 8° | 8° | 8° | 15° | 8° | 12° |
| Matrix | 256×256×176 | 256×256×176 | 256×256×176 | 256×256×176 | 256×231×170 | 256×256×170 | 256×256×182 | 256×256×124 | 256×256×180 | 256×256×176 |
| Manufacturer, model | 3.0T Siemens Trio | 3.0T Siemens Trio | 3.0T Siemens Trio | 1.5 T GE magnet | 3.0T Phillips Intera | 3.0T Phillips Intera | 3.0T Phillips Intera | 1.5 T Signa Excite system | 3.0T Phillips Intera | 3.0T Siemens Trio |
| **Treatment seeking** | outpatients (with & without treatment seeking) | outpatients (with & without treatment seeking) | inpatients | inpatients | outpatients | outpatients (treatment seeking) | - | - | - | - |
| **Substance use measures** |  |  |  |  |  |  |  |  |  |  |
| Pre-MRI abstinence | 24 h | 24 h | 11-17 days (mean:15 days) | 24 h | 24 h | ≥ 15 days (mean:18 days) |  |  |  |  |
| Severity of AUD | AUDIT, TLFB | AUDIT |  | TLFB | AUDIT | AUDIT |  |  |  |  |
| Standard drinks/month, N | yes | yes | yes | yes | yes | yes | yes | yes | yes | yes |
| Cigarettes/month, N | yes | yes | yes | yes | yes | yes | no | yes | yes | yes |

AUD: Alcohol use disorder; SCID: Structured Clinical Interview for DSM Disorders (Spitzer et al. 1994; First et al. 2001); MINI: Mini Neuropsychiatry International Interview (Lecrubier et al. 1997; Sheehan et al. 1997; Swift et al. 1998); CIDI: International Diagnostic interview (CIDI; Robins et al. 1988); PRISM = Psychiatric Research Interview for Substance and Mental Disorders (http://www.columbia.edu/~dsh2/prism/, Hasin et al. 1996); AUDIT = Alcohol Use Disorders Identification Test (Saunders et al. 1993); TLFB = Time-line Follow-back (Sobell and Sobell, 1992). StDr= standard drinks; Cig= cigarettes; mo=month.

**Supplementary references**

Catani, M., & Thiebaut de Schotten, M. (2008). A diffusion tensor imaging tractography atlas for virtual in vivo dissections. *Cortex, 44*(8), 1105-1132. doi:10.1016/j.cortex.2008.05.004

Grodin, E. N., Lin, H., Durkee, C. A., Hommer, D. W., & Momenan, R. (2013). Deficits in cortical, diencephalic and midbrain gray matter in alcoholism measured by VBM: Effects of co-morbid substance abuse. *NeuroImage: Clinical, 2*, 469-476.

Momenan, R., Steckler, L. E., Saad, Z. S., van Rafelghem, S., Kerich, M. J., & Hommer, D. W. (2012). Effects of alcohol dependence on cortical thickness as determined by magnetic resonance imaging. *Psychiatry Research: Neuroimaging, 204*(2-3), 101-111.

Senatorov, V. V., Damadzic, R., Mann, C. L., Schwandt, M. L., George, D. T., Hommer, D. W., . . . Momenan, R. (2015). Reduced anterior insula, enlarged amygdala in alcoholism and associated depleted von Economo neurons. *Brain, 138*(1), 69-79.

Tzourio-Mazoyer, N., Landeau, B., Papathanassiou, D., Crivello, F., Etard, O., Delcroix, N., . . . Joliot, M. (2002). Automated anatomical labeling of activations in SPM using a macroscopic anatomical parcellation of the MNI MRI single-subject brain. *Neuroimage, 15*(1), 273-289. doi:10.1006/nimg.2001.0978

van Holst, R. J., Clark, L., Veltman, D. J., van den Brink, W., & Goudriaan, A. E. (2014). Enhanced striatal responses during expectancy coding in alcohol dependence. *Drug and alcohol dependence, 142*, 204-208.
